# All in the family: a population-scale perspective on genetic networks of autoimmune disease

**DOI:** 10.1172/JCI211202

**Published:** 2026-10-01

**Authors:** Arielle Klepper, Mark S. Anderson

**Affiliations:** 1 Department of Medicine,; 2 Liver Center, and; 3 Diabetes Center, UCSF, San Francisco, California, USA.

## Abstract

Autoimmune diseases share a genetic predisposition, and GWAS have identified certain HLA haplotypes as components of susceptibility. In this issue of the *JCI*, Eriksson et al. leveraged population-scale registry data from Sweden, encompassing 6.3 million individuals in combination with paired relatedness data from a remarkable 3.84 million sibling pairs drawn from 1.57 million nuclear families to create a unified model of the shared genetic risk across 22 common and rare autoimmune diseases. The resulting network of pairwise genetic relationships demonstrated that genetic predisposition alone could not explain the full spectrum of autoimmunity. Analyses exposed distinct patterns, confirmed expected genetic relationships, and revealed unexpected findings. By looking into the family to quantify shared genetic liability at a remarkable scale, this study not only reinforces established genetic components of autoimmune disease but also highlights variation that lacks shared genetic architecture, paving the way for future study of environmental mechanisms in autoimmunity.

## Introduction

Autoimmune diseases represent a significant clinical burden, with estimates of 5%–10% of the population being affected (1). Well over 100 autoimmune conditions have been described, and they are thought to arise through a breakdown in immune self-tolerance (2). The etiology of this breakdown is complex and involves a mixture of both environmental and genetic factors. Studies of certain autoimmune conditions involving identical twins have established a concordance rate that is higher than that for siblings or the conditions’ prevalences in the general population, which helps underscore the genetic component to disease. At the same time, it is important to note that for any given autoimmune condition, the identical twin lifetime concordance rate is most commonly below 50%. This modest concordance rate reveals the permissive genetics of autoimmune disease, reflecting the stochastic nature of adaptive immune system generation as well as the influence of environmental factors in this process.

Much of the genetic work in recent years has focused on identifying common genetic variants that are linked to single, more common autoimmune conditions through approaches like GWAS. Most GWAS have highlighted the HLA locus as a major component of genetic susceptibility, along with a host of other common variants in various immune gene pathways with weaker influence. The HLA (human leukocyte antigen) locus is a highly polymorphic region of the human genome and comprises a large number of immunity-related genes that include the class I and class II MHC genes. Low recombination rates across the HLA locus lead to blocks of alleles that cluster together into heritable units called HLA haplotypes, some of which are referred to as “risk haplotypes” due to their association with disease. Despite advances in understanding genetic contributions to autoimmunity, examination of autoimmune disease sharing patterns in siblings across both common and rare autoimmune disease features has been somewhat understudied. In this issue of the *JCI*, in an extremely large cohort study from northern Europe, a team of investigators leveraged the large numbers of patients tracked over time to examine disease sharing patterns for a host of autoimmune diseases.

## Zooming out: population scale enables fresh genetic insights

The approach used by Eriksson et al. (3) demonstrates the principle of strength in numbers. While autoimmune diseases may be individually rare — e.g., Addison’s disease impacted only 0.02% of the queried population in this study — in aggregate, they impact huge numbers of people. Specifically, this study observed that autoimmune disease impacted 11.2% of the Swedish population cohort (707,995 individuals), investigated at population scale (6.3 million subjects), including 15.3% of women and 7.2% of men. These figures are remarkably consistent with population-level data from the United Kingdom, where autoimmune diseases affected 10.2% of the population (13.1% of women and 7.4% of men) (1), further underscoring both the substantial burden of autoimmunity, its predominance in women, and the power of studying rare and common autoimmune diseases collectively.

While individual subspecialty clinics may see a narrow scope of autoimmune diseases, this study has a key advantage of being able to zoom out to study population-wide disease, allowing a reorientation of perspective to encompass 22 autoimmune conditions. By pairing patient data with widespread registry data and a full sibling registry, comprising 3,839,400 full sibling pairs from 1,574,407 nuclear families, Eriksson et al.’s approach (Figure 1) enables large-scale analysis of shared inherited susceptibility. Using population-level data, along with sibling pair data, the authors constructed a matrix of pairwise genetic correlations. This profound methodological advance builds on prior instrumental population-scale studies (1, 4) to view genetic relationships in autoimmune disease in aggregate. This approach was also markedly less constrained by the number of participants, a common challenge faced by GWAS examining individual or related groups of autoimmune diseases.

## Family ties revisited

Prior studies of families have established two foundational principles about the genetic patterns in autoimmune disease. First, individual autoimmune diseases exhibit strong familial aggregation, supporting an important inherited component to disease susceptibility (5–7). Population-scale familial aggregation has been observed for multiple sclerosis, type 1 diabetes, psoriasis, and Crohn’s disease (4). While the prior work has identified some cross-disease familial associations, much weaker clustering was observed between different autoimmune diseases within families than within the same disease. However, there are known examples of shared genetic susceptibility, such as celiac disease and type 1 diabetes, which share HLA-DQ2 and HLA-DQ8 risk haplotypes. Subsequent population-scale analyses from the United Kingdom have found that different autoimmune diseases do not co-occur at random, but tend to aggregate in recognizable clinical patterns (1). Notably, connective tissue diseases clustered together, while endocrine-related autoimmune diseases, including type 1 diabetes, autoimmune thyroid disease, and Addison’s disease, formed a second major cluster that also included celiac disease. However, while measures of familial aggregation illustrate important epidemiologic patterns, pairwise comparisons cannot readily describe relationships across the full spectrum of autoimmune disease.

The major conceptual advance of Eriksson et al.’s work is that the authors moved beyond comparing autoimmune diseases one pair at a time, but instead estimated the shared susceptibility among all 22 autoimmune diseases simultaneously (3). This approach of allowing 22 different autoimmune diseases to cluster naturally into relatedness modules revealed relationships that were not apparent from individual pairwise comparisons.

The inclusion of genetic correlations allowed the authors to model one fundamental question: can underlying genetic risk (e.g., HLA type) explain all the observed variability in autoimmune disease? Unsurprisingly, given what we already know, Eriksson et al. could not identify a singular driving genetic liability. The authors performed principal component analysis on the genetic correlation matrix and identified that the first principle component, PC1, explained only 21% of the variance, and it took six PCs to explain approximately two-thirds of the variance. Thus, inherited susceptibility alone cannot be reduced to a single “autoimmune axis,” suggesting that disease-specific biology — and likely environmental influences — remain major contributors to autoimmune disease pathogenesis.

Further investigation of the clusters across the correlation matrix of the 22 autoimmune diseases studied revealed both expected and unexpected patterns. Eriksson et al. were able to demonstrate familial correlations across different autoimmune diseases, and these patterns clustered frequently along tissue lines. Consistent with clinical experience, there were expected correlations between psoriasis and psoriatic arthritis, liver-related autoimmune diseases autoimmune hepatitis and primary biliary cholangitis, and connective tissue diseases such as systemic lupus erythematosus and Sjögren’s syndrome. Among the expected findings, celiac disease and dermatitis herpetiformis demonstrated the strongest familial association and strong shared inherited susceptibility, consistent with their shared HLA-DQ2/DQ8–mediated biology. However, network analysis revealed that dermatitis herpetiformis had many more interaction partners within the autoimmune disease network, raising the possibility that beyond gluten reactivity, dermatitis herpetiformis could be associated with a more pleiotropic set of inherited risk factors.

## Unexpected gaps in genetic clustering encourage further investigation

Regarding unexpected findings, there was notably no clustering between type 1 diabetes and psoriasis, suggesting that common autoimmune diseases do not necessarily share a common inherited architecture. This is despite the well-described co-occurrence between psoriasis and type 2 diabetes, which is largely attributed to shared metabolic risk factors such as obesity rather than autoimmunity. Furthermore, multiple sclerosis again emerged as an outlier. Despite its previously reported strong familial aggregation (4, 6), multiple sclerosis exhibited only weak shared inherited susceptibility with most other autoimmune diseases. This distinction underscores that even highly heritable diseases may still have genetically distinct origins. The authors also note that multiple sclerosis demonstrated little shared inherited susceptibility with the other neurologic autoimmune disease included in the analysis, myasthenia gravis, suggesting that neurologic autoimmune diseases do not form a distinct inherited cluster in the same way as liver, connective tissue, or endocrine autoimmune diseases.

In contrast to clinical expectations, two common autoimmune thyroid diseases, Graves’ disease and Hashimoto’s thyroiditis, demonstrated weaker shared inherited susceptibility than anticipated. Despite being clinically grouped together as autoimmune thyroid disease, the two diseases were not more genetically related than many other autoimmune disease pairs, suggesting that these clinically similar disorders may arise through more distinct genetic pathways than was previously appreciated. Appendicitis, included as a negative control in this study, was negatively associated with ulcerative colitis, consistent with long-standing epidemiologic observations that appendectomy is associated with a reduced risk of ulcerative colitis (8, 9). Also notable was that primary sclerosing cholangitis (PSC), a chronic inflammatory disease of the liver and biliary tree, demonstrated stronger shared inherited susceptibility with inflammatory bowel disease than with the other autoimmune liver diseases. Given the well-established clinical association between PSC and inflammatory bowel disease, this finding is particularly intriguing and suggests that PSC may share pathogenic mechanisms with inflammatory bowel disease that extend beyond classical autoimmune pathways, highlighting an important area for future mechanistic investigation.

## Looking ahead: where do autoimmune networks lead?

Eriksson et al. have enabled a large-scale view of autoimmunity. This vantage point recapitulated tissue-specific disease groupings upon which we rely clinically. These relationships suggest that, for highly correlated diseases, future GWAS that include related autoimmune diseases together may have the potential to uncover shared genetic loci and may be able to recruit larger numbers of patients to enable identification of rare mutations, influencing future study design. The absence of strong correlations in this study was as informative as the correlations that were identified. Although there were patterns of shared susceptibility by tissue types, there was also the surprising finding that two common thyroid autoimmune diseases have only modest sharing. Furthermore, multiple sclerosis remained distinct. While inherited risk clearly contributes to multiple sclerosis, these findings suggest that disease-specific biology — or additional environmental triggers, such as Epstein-Barr virus infection — may play a comparatively greater role than in many other autoimmune diseases.

This study does have notable limitations. As the authors note, because sibling pairs often share environmental exposures, the liability-threshold model cannot fully distinguish shared environmental effects from inherited susceptibility, potentially leading to overestimation of some genetic associations. Finally, improving our understanding of shared inherited susceptibility across autoimmune diseases may refine disease classification, improve screening and diagnosis, and ultimately create opportunities for disease prevention and screening when available.

## Conflict of interest

MSA reports ownership of stock in Merck and Medtronic and is a consultant for Cour Pharmaceuticals.

## Funding support

This work is the result of NIH funding, in whole or in part, and is subject to the NIH Public Access Policy. Through acceptance of this federal funding, the NIH has been given a right to make the work publicly available in PubMed Central.

- NIH grant K08DK139453 (AK).
- NIH grants P30DK135103, R01DK137904, and U01DK143494 (MSA).
- National Institute of Allergy, Immunology, and Infectious Disease of the NIH grants R21AI191139, P01AI5539, R37AI97457, and UM1AI109565 (MSA).
- Helmsley Charitable Trust (MSA).
- Northern California Center of Excellence for Breakthrough T1D (MSA).
- Chan Zuckerberg Biohub Investigator (MSA).

## Figures and Tables

**Figure 1:**
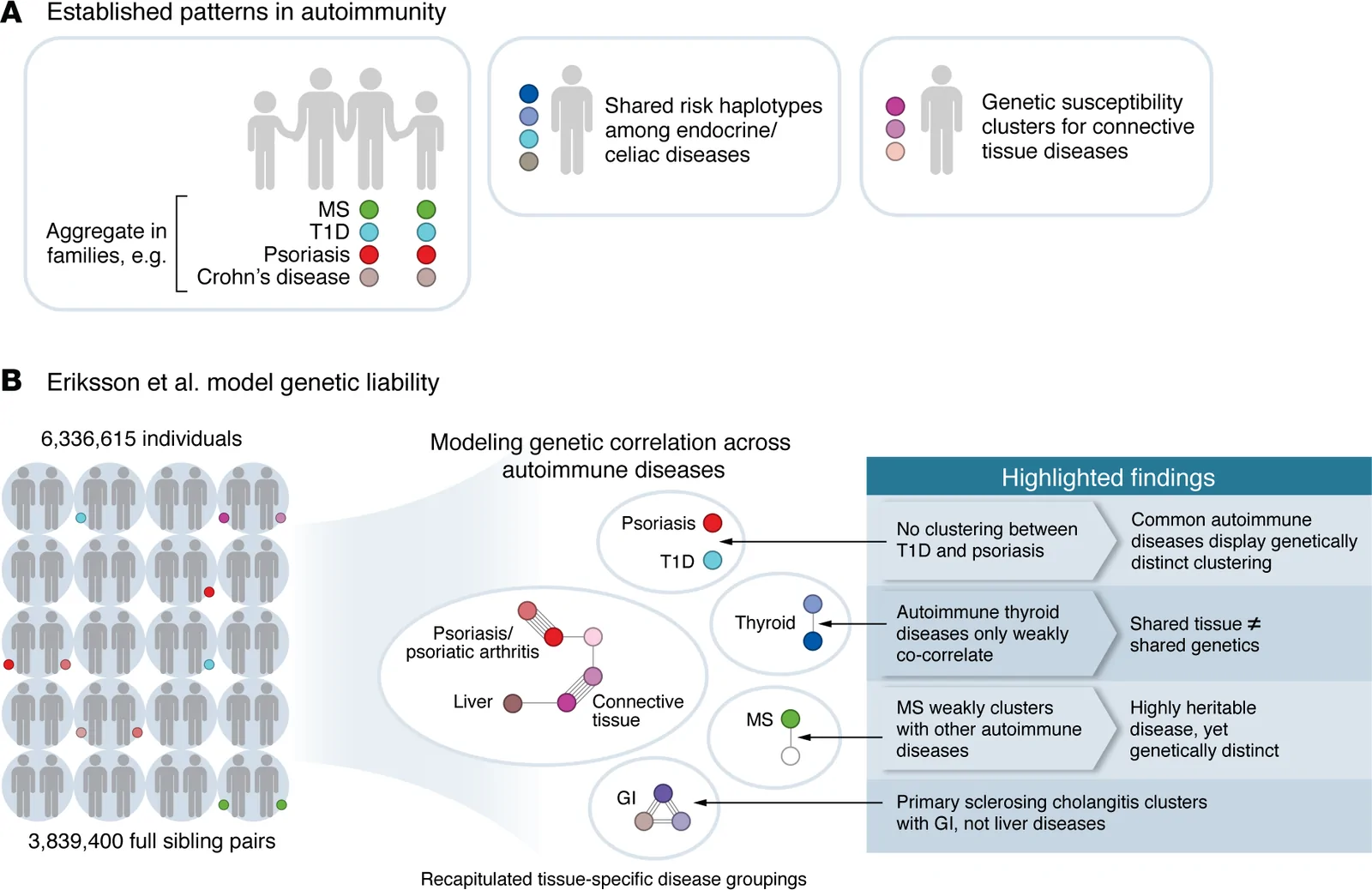
Genetic patterns underlying autoimmune diseases. (**A**) Established patterns: some diseases, such as multiple sclerosis (MS) and type 1 diabetes (T1D), aggregate in families (left). Other autoimmune diseases are known to aggregate in individuals, in part due to shared genetic susceptibility, such as HLA variants common to both T1D and celiac disease (center), or clustering of connective tissue diseases in individuals, e.g., systemic lupus erythematosus and Sjögren’s syndrome (right). (**B**) Eriksson et al. (3) compared susceptibility across 22 autoimmune diseases in a large Swedish cohort, leveraging sibling registry data to construct a matrix of pairwise genetic correlations. Their findings confirm some prior insights about genetic patterns, such as the clustering of tissue-specific diseases, but they also unearth fresh insights into variation among autoimmune diseases, as summarized in the highlighted findings. GI, gastrointestinal.

## Version 1 10/01/2026

Electronic publication
